# Population genetic analysis of aquaculture salmonid populations in China using a 57K rainbow trout SNP array

**DOI:** 10.1371/journal.pone.0202582

**Published:** 2018-08-17

**Authors:** Han-Yuan Zhang, Zi-Xia Zhao, Jian Xu, Peng Xu, Qing-Li Bai, Shi-Yong Yang, Li-Kun Jiang, Bao-Hua Chen

**Affiliations:** 1 Key Laboratory of Aquatic Genomics, Ministry of Agriculture, Beijing Key Laboratory of Fishery Biotechnology, Chinese Academy of Fishery Sciences, Beijing, China; 2 Fujian Collaborative Innovation Center for Exploitation and Utilization of Marine Biological Resources, College of Ocean and Earth Sciences, Xiamen University, Xiamen, China; 3 Heilongjiang River Fisheries Research Institute, Chinese Academy of Fishery Sciences, Harbin, China; 4 College of Animal Science and Technology, Sichuan Agricultural University, Yaan, China; National Cheng Kung University, TAIWAN

## Abstract

Various salmonid species are cultivated in cold water aquaculture. However, due to limited genomic data resources, specific high-throughput genotyping tools are not available to many of the salmonid species. In this study, a 57K single nucleotide polymorphism (SNP) array for rainbow trout (*Oncorhynchus mykiss*) was utilized to detect polymorphisms in seven salmonid species, including *Hucho taimen*, *Oncorhynchus masou*, *Salvelinus fontinalis*, *Brachymystax lenok*, *Salvelinus leucomaenis*, *O*. *kisutch*, and *O*. *mykiss*. The number of polymorphic markers per population ranged from 3,844 (*O*. *kisutch*) to 53,734 (*O*. *mykiss*), indicating that the rainbow trout SNP array was applicable as a universal genotyping tool for other salmonid species. Among the six other salmonid populations from four genera, 28,882 SNPs were shared, whereas 525 SNPs were polymorphic in all four genera. The genetic diversity and population relationships of the seven salmonid species were studied by principal component analysis (PCA). The phylogenetic relationships among populations were analyzed using the maximum likelihood method, which indicated that the shared SNP markers provide reliable genomic information for population genetic analyses in common aquaculture salmonid fishes. Furthermore, this obtained genomic information may be applicable for population genetic evaluation, marker-assisted breeding, and propagative parent selection in fry production.

## Introduction

Salmonid fishes are naturally distributed in freshwater and marine habitats around the world. Salmonid fishes are of high economic, recreational, and ecological value. However, the genetic variation of salmonid species has decreased to different degrees because of increased inbreeding due to human activities. For aquaculture species, supportive or assistant breeding causes inbreeding depression or loss of genetic variability [1,2]. Inbreeding can occur at any stage of the hatchery or broodstock such as breeding among close relatives, low numbers of broodstock, systematic selection for specific traits, and unequal mortality of offspring among families [3,4,5]. Inbreeding depression in salmonids is the main effect of inbreeding in conservation and aquaculture, which in turn reduces heterozygosity as well as the mean value of fitness-related phenotype. Maintaining genetic diversity within and among populations is thus a high priority in salmonid conservation and aquaculture [6,7]. An understanding of the population genetic structure of salmonid populations is essential for fisheries resource conservation and management.

The family Salmonidae consists of three subfamilies and 11 genera, including more than 70 species [8]. Species in genera *Oncorhynchus*, *Salvelinus*, *Brachymystax*, and *Hucho* are major cold water cultural fishes in China. Among these salmonid fishes, rainbow trout, *O*. *mykiss*, is the most widely cultivated species. It is also a worldwide aquaculture species with relatively well investigated resources of genomic data [9–12]. Based on the whole genome shotgun strategy, the first version of rainbow trout reference genome was released in 2014, representing a 1.9-Gb genome assembly with a scaffold N50 of 384 kb [13]. Abundant single nucleotide polymorphism (SNP) markers were identified by genome library sequencing [14], RNA-seq [15,16], expressed sequence tags (EST) sequencing [17], restriction-site associated DNA (RAD) sequencing [18], and genome resequencing [19]. Out of approximately 2.12 M candidate SNPs, a commercially available 57K high-density SNP array for rainbow trout was developed in 2015 [19]. This array contained 50,701 high-quality SNPs with a wide distribution and good representation throughout the genome. This SNP array was successfully used in population genetic analysis [20], genome-wide association study (GWAS) [21], and genomic selection in rainbow trout breeding [22].

However, except for rainbow trout and Atlantic salmon (*Salmo salar*), two salmonid fishes with whole genome sequencing projects, genomic resources for other salmonid fishes are limited. Mitochondrial DNA and microsatellites have thus been used as resources for genetic analysis of other salmonid species [23–26]. In a previous attempt to develop and evaluate SNP arrays for common carp (*Cyprinus carpio*) we observed that a considerable number of polymorphic SNPs are shared among phylogenetically related species. Thus, the common carp SNP array may be utilized in eight other species of family Cyprinidae [27]. In addition, it might be practical to use the rainbow trout SNP array as a universal genetic tool for the genetic analysis of other salmonid species, which is more efficient and economical in sequencing and large-scale marker discovery for each species.

In this study, the 57K rainbow trout SNP array was used for genotyping 96 individuals representing seven salmonid species to assess genetic diversity and population structure of different populations. The call rate quality and shared SNPs among species were analyzed to provide universal polymorphic molecular markers for future genetic analysis and genomic selection in salmonid fish breeding.

## Materials and methods

### Fish and DNA samples

In this study, 96 cultured samples representing seven salmonid species were collected for genotyping and further analyses. Fish samples were collected at three sites: Mudanjiang in Heilongjiang, Dujiangyan in Sichuan, and Huairou in Beijing, China. Unrelated individuals from seven salmonid species were sampled: *Hucho taimen* (HT, sample numbers 1–8), *Oncorhynchus masou* (OM, sample nos. 9–16), *Salvelinus fontinalis* (SF, sample nos. 17–24), *Brachymystax lenok* (BL, sample nos. 25–32), *Salvelinus leucomaenis* (SL, sample nos. 33–40), *Oncorhynchus kisutch* (OK, sample nos. 41–48), and *Oncorhynchus mykiss* rainbow trout (RT, sample nos. 49–96). All sampling procedures complied with the guidelines of Animal Care and Use Committee (ACUC) of the Centre for Applied Aquatic Genomics, Chinese Academy of Fishery Sciences. All sampling procedures and experimental manipulations were approved as part of obtaining the field permit. Approximately 1 cm^2^ tail fin tissue was cut from each individual, and then dried at 56 ^o^C before short storage at room temperature. The sampled individuals were released to the ponds for natural fin regeneration. Genomic DNA was extracted from fin tissues using a Marine Animal Genomic DNA Extraction Kit (TIANGEN, Beijing), following the manufacturer’s recommendations. The integrity of DNA was analyzed on 1% agarose gels. DNA was quantified using a NanoDrop 8000 device (Thermo Scientific, USA).

### SNP genotyping and validation

More than 5,000 ng of genomic DNA from each sample were sent to a commercial service provider (Geneseek, Inc., Lincoln, NE, USA) for genotyping, which was performed using the 57K rainbow trout SNP array, according to Axiom genotyping procedures described by Affymetrix. Affymetrix Power Tools (APT) and SNPOLISHER software packages were used for genotyping and quality control, following the manufacturer’s recommendations (http://media.affymetrix.com/support/downloads/manuals/ axiom_best_practice_supplement_user_guide.pdf). SNP validation and statistics was processed using PLINK v1.09 (https://www.cog-genomics.org/plink2) [28,29]. A moderate call rate threshold (>85%) was set for SNP calling. SNPs with no less than two genotypes within one population and higher than 5% minor allele frequency (MAF) were considered as polymorphic SNPs.

### Population structure analysis

Principal component analysis (PCA) was performed to assess the genetic relationships among seven salmonid species using PLINK v1.09 and GCTA (Genome-wide Complex Trait Analysis) software [30], respectively. Different datasets of SNPs with varying numbers of markers ranging from 2,393 to 57,501 were used to verify the consistency of the results, with different filter thresholds: 1) 57,501 SNPs with no filter; 2) 53,436 SNPs with a call rate >85% for all 96 individuals; 3) 28,882 SNPs with call rate >85% in each population; and 4) 2,393 SNPs with a call rate >85% in each population and MAF >5% in each population. Linkage disequilibrium (LD) and allele frequency analysis were performed with PLINK v1.09. The filter threshold for LD and allele frequencies was r^2^ < 0.2 and MAF > 0.02, respectively. The R package (www.r-project.org/) was used to visualize the PCA results. Customized high-resolution Venn diagrams were constructed to visualize the shared and unique SNPs among the four salmonid genera using the VennDiagram suite in the R package [31]. The distribution and relationship of SNPs among *Hucho*, *Oncorhynchus*, *Salvelinus*, and *Brachymystax* genera were evaluated. One species from each genus was selected as representative.

### Phylogenetic analysis

Phylogenetic analysis of the salmonid populations was performed with CLC Genomics Workbench 9.5.4, and a maximum likelihood phylogenetic tree was generated. Bootstrapping was performed using the group population option with 1,000 replicates over loci. Phylogenetic analysis was conducted using the genotyping data of 11,643 SNPs (call rate >99%) shared by all populations. A phylogenetic tree was constructed using EvolView v2 [32,33].

## Results

### SNP genotyping and validation

The 96 salmonid individuals used in this study represented seven different populations based on species and strains, namely, HT, OM, SF, BL, SL, OK, and RT. The call rate distribution of each sample is shown in S1 Fig, indicating that the call rate of non-rainbow trout samples ranged from 90.621% to 93.339%, which was much lower than that of rainbow trout samples with high genotyping quality (call rate as high as 99.078%).

While a moderate call rate threshold was set (>85%), all the non-rainbow trout samples passed quality control. The number of informative SNPs in the seven populations varied from 47,347 to 55,955. The number of polymorphic SNPs in each group ranged from 3,844 in the OK population to 53,734 in the RT population. The RT population exhibited a high SNP polymorphic rate (96%). Although belonging to the same genus *Oncorhynchus*, the OK and OM populations showed low polymorphic rates (7.8% and 13.8%, respectively). The number of SNPs and polymorphic SNPs of each population are summarized in Table 1.

**Table 1:** Summary of polymorphic SNPs in seven cultured salmonid populations.

Species	Sample ID	Sampling site	Number of genotyped SNPs	Percentage of genotyped SNPs (%)	Number of polymorphic SNPs	Percentage of polymorphic SNPs (%)
*Hucho taimen*	1~8	Mudanjiang, Heilongjiang, China	47,418	82.5	6,838	14.4
*Oncorhynchus masou*	9~16	Mudanjiang, Heilongjiang, China	48,401	84.2	6,699	13.8
*Salvelinus fontinalis*	17~24	Mudanjiang, Heilongjiang, China	47,723	83.0	17,442	36.5
*Brachymystax lenok*	25~32	Mudanjiang, Heilongjiang, China	47,347	82.3	18,154	38.3
*Salvelinus leucomaenis*	33~40	Mudanjiang, Heilongjiang, China	47,516	82.6	6,650	14.0
*Oncorhynchus kisutch*	41~48	Dujiangyan, Sichuan, China	49,100	85.4	3,844	7.83
*Oncorhynchus mykiss*	49~96	Mudanjiang, Heilongjiang, China	55,955	97.3	53,734	96.0
		Dujiangyan, Sichuan, China				
		Huairou, Beijing, China				

**Fig 1:**
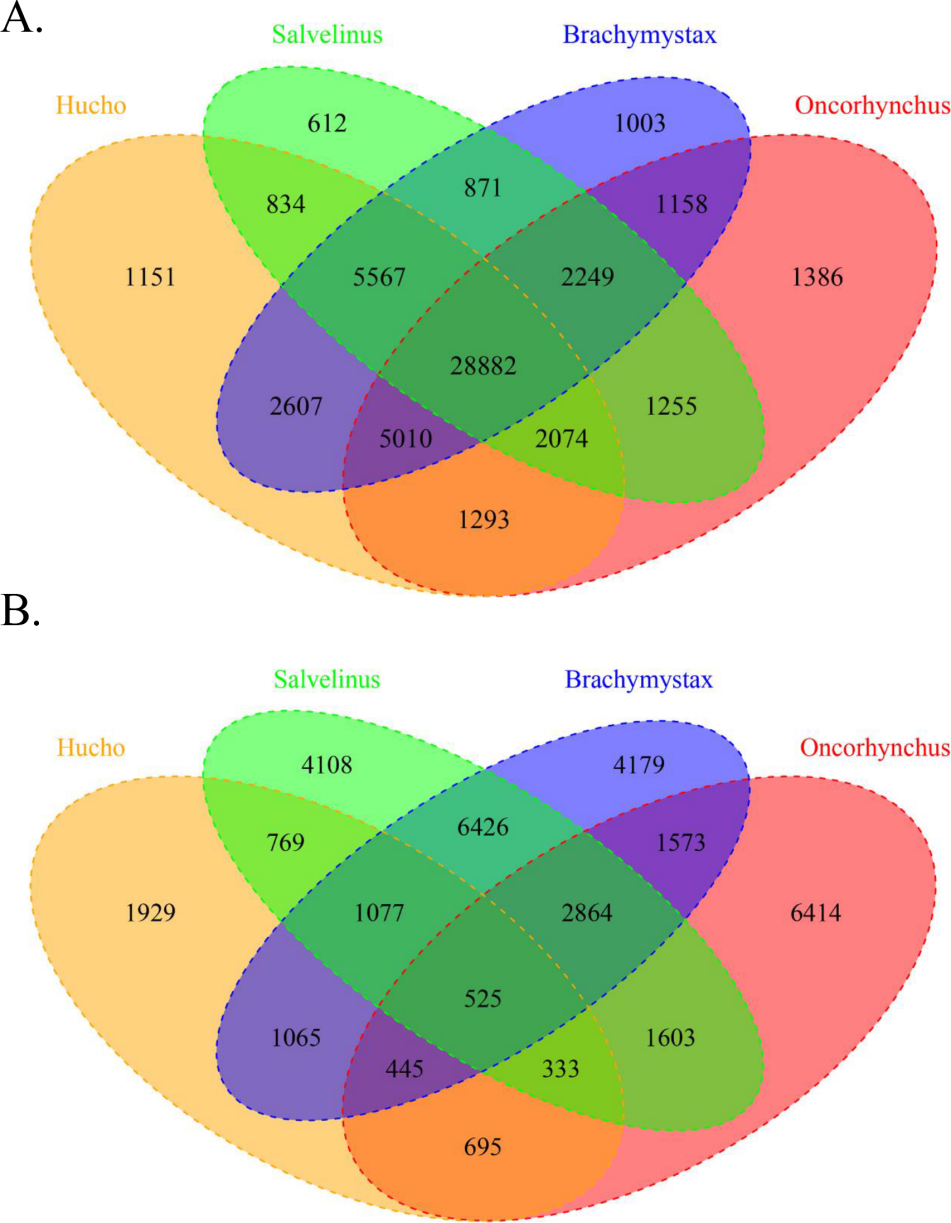
The four-set Venn diagrams of four salmonid genera. Each genus is represented by one color: yellow, *Hucho*; red, *Oncorhynchus*; green, *Salvelinus*; and blue, *Brachymystax*. The number of shared and unique SNPs is shown in different blocks. (A) The distribution of shared and unique informative SNPs and (B) the distribution of shared and unique polymorphic SNPs among the four genera.

The shared SNP statistics of the four genera are presented using Venn diagrams (Fig 1). In terms of informative SNPs (Fig 1A), the number of specific SNPs in the *Hucho*, *Oncorhynchus*, *Salvelinus*, and *Brachymystax* genera were 1,151, 1,386, 612, and 1,003, respectively. In contrast, in terms of polymorphic SNP sets (Fig 1B), by contrast, four genera showed more specific SNPs (*Hucho*: 1,929; *Oncorhynchus*: 6,414; *Salvelinus*: 4,108; and *Brachymystax*: 4,179). The number of informative and polymorphic SNPs in the overlapping area of the Venn diagram among the four genera was 28,882 and 525, respectively. *Hucho* and *Brachymystax* shared the highest number of SNPs (42,066) between two genera, whereas *Salvelinus* and *Brachymystax* shared the highest number of polymorphic SNPs (10,892) between two genera.

### Population diversity and genetic relationships among different salmonid populations

The genetic relationships among salmonid individuals and populations were analyzed by PCA. Different SNP datasets were obtained by different filter thresholds: 1) 57,501 SNPs with no filter; 2) 53,436 SNPs with a call rate >85% for all 96 individuals; 3) 28,882 SNPs with call rate >85% in each population; and 4) 2,393 SNPs with a call rate >85% in each population and MAF >5% in each population. PCA of each dataset was performed using PLINK v1.09 and GCTA. With varied principal component (PC) values, the clustering distributions of individuals were highly similar among the PCA results while using 1) - 3) datasets and both software. The 96 individuals were clustered into four groups: 48 rainbow trout individuals were clustered as a loose group, and the other six species were classified into three separate groups. While the number of SNPs was reduced to 2,393, all the non-rainbow trout individuals belonged to a single group.

**Fig 2:**
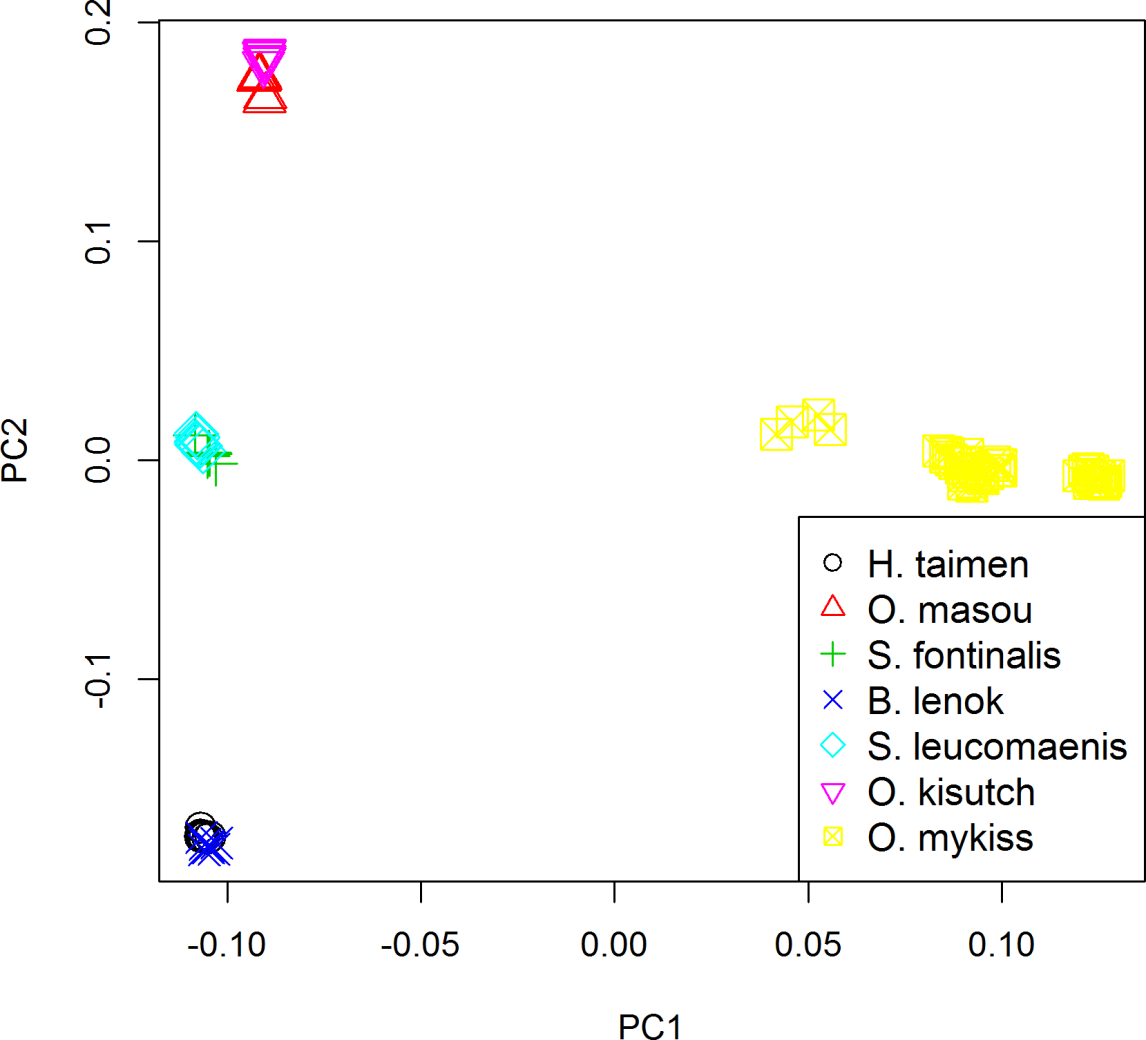
PCA of 96 individuals from seven salmonid populations (PC1: 33.4% of variance against PC2: 8.2% of variance) based on 28,882 shared SNP markers using the GCTA software. Each individual is represented by one point. Each population is shown by different symbols.

The GCTA results of 28,882 SNPs was adopted in this study (Fig 2). In the analysis of the 96 individuals from seven salmonid populations, eight PC factors occupied at least 2% of the observed variations by each factor. A total of 65.1% of genetic variations among populations could be explained by these eight PC factors. PC1 to PC4 determined 33.4%, 8.2%, 7.1%, and 5.5% of the variations, respectively. PC1 and PC2 differentiated *Oncorhynchus mykiss* from the other six populations. The HT and BL populations were clustered together, whereas the OM and OK populations and the SF and SL populations were respectively clustered into one group.

### Phylogenetic analysis

**Fig 3:**
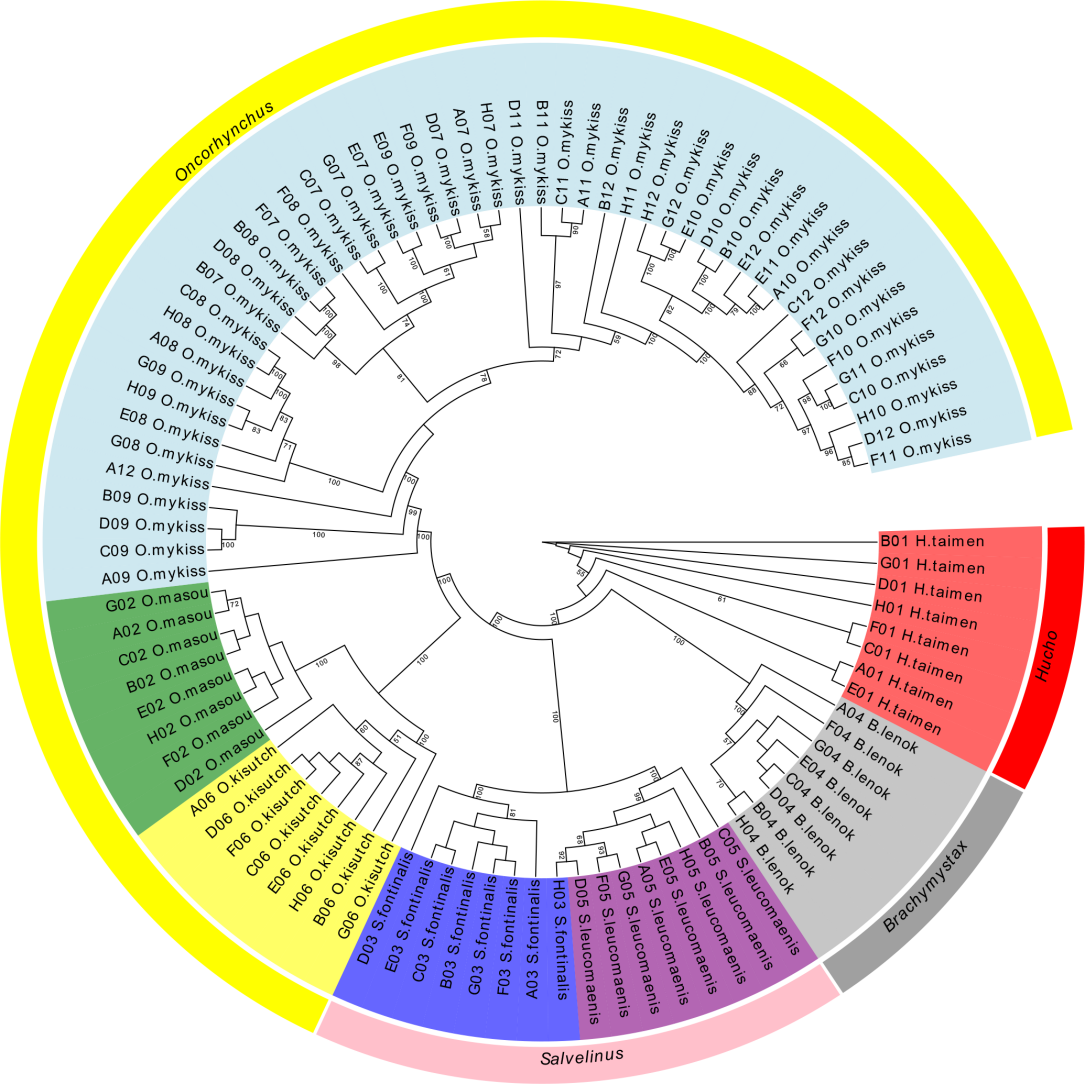
Phylogenetic tree of 96 individuals from seven salmonid populations that was constructed using the maximum likelihood method. The inner circle shows seven salmonid populations: red, *H*. *taimen*; grey, *B*. *lenok*; purple, *S*. *leucomaenis*; blue, *S*. *fontinalis*; yellow, *O*. *kisutch*; green, *O*. *masou*; and light blue, *O*. *mykiss*. The outer circle represents four corresponding genera: red, *Hucho*; grey, *Brachymystax*; pink, *Salvelinus*; and yellow, *Oncorhynchus*. The confidence level of 1,000 bootstrap replications is shown at the branch nodes.

Phylogenetic reconstruction of the 96 individuals representing seven salmonid populations was performed using a dataset of high-genotyping quality 11,643 SNPs, which were obtained using a call rate threshold of >99%. The seven species were clearly differentiated in the maximum likelihood tree (Fig 3), with high bootstrap values on the branches. The clade distributions of the samples were coincided with their taxonomic classifications. Forty-eight rainbow trout individuals were grouped as one big clade, and the genetic distances were significantly longer than the other clades. The OM and OK clades, which also belonged to genus *Oncorhynchus*, were clustered with the *O*. *mykiss* clade at one branch. A parallel branch consisting of the SL clade and clades were classified under genus *Salvelinus*.

## Discussion

Comparative genomic studies have indicated that salmonid fishes emerged after a whole genome duplication event involving an autotetraploid ancestor, which was estimated to have occurred 88–103 million years ago [13,34–37]. The genomes of these species have returned to its stable diploid state mainly through chromosomal rearrangements and divergence of homologous chromosomes, while the DNA sequences remain highly similar, and a lot of molecular markers are shared among the salmonid fishes [24,26]. The rate of loss of heterozygosity in the tetrasomic loci of these salmonids decreased, thereby resulting in inbreeding [38], indicating that these species have higher sequence similarity compared to diploid fishes [39]. The present study observed high call rates (all >90%) for the rainbow trout SNPs in non-rainbow trout individuals, thereby confirming the hypothesis of high sequence similarity. The percentages of shared SNPs among salmonid species, which are approximately 82.3% to 85.4%, were significantly higher than those of cyprinid fishes [27], which ranged from 11.9% to 23.6%.

The shared SNPs varied among non-rainbow trout populations, indicating that these markers may be utilized for genetic analyses. With eight individuals sampled in each population, polymorphic rates of 7.8% (OK) to 38.3% (BL) were observed. These observations suggest that the aquaculture population of *B*. *lenok*, a native salmonid species in China, exhibits high genetic diversity. However, some aquaculture species that were introduced from foreign countries, which include *O*. *kisutch*, showed limited polymorphisms, suggesting that their genetic diversity is low, and introduction of new broodstock is necessary.

The 57K SNP array has been utilized in three non-rainbow trout species namely *O*. *clarkii* (N = 5), *O*. *tshawytscha* (N = 3), and *O*. *kisutch* (N = 4) [19]. A relatively high number of shared SNPs was obtained (33-47K), but only 6.25%-15.8% shared SNPs were found to be polymorphic. Comparing to the present study, the observed smaller number of shared SNPs might be due to our use of a strict call rate threshold (>97%), and the lower percentages of polymorphic SNPs may have been caused by the small size of the study population. Thus, the practicability of the SNP array in related species is apparently better than the previous estimates.

PCA is a dimension-reducing technique that represents all datasets with a handful of SNPs corresponding to the most significant PCs and can be used to visualize population relationships. As shown in our results, a large dataset (28,882 shared SNPs) was necessary to extensively illustrate the genetic diversity and population relationships among representative salmonid populations in China (Fig 2). The non-rainbow trout individuals were clustered based on genus, except for the HT and BL populations, which were tightly clustered together. In addition, the rainbow trout populations apparently exhibited stratification, and at least three subgroups were observed. Forty-eight rainbow trout were collected from three different sampling sites, including 16 fishes per sampling site. The results of PCA reflected the differences among various strains. The point distributions were highly consistent with the genetic distances shown in the phylogenetic tree.

The phylogenetic tree was constructed based on both polymorphic and monomorphic SNPs, with the latter because providing essential information on conserved sites. The positions of different species in the phylogenetic tree coincide with their evolutionary relationships, indicating that the genotyping results are highly reliable. The tree branch of *O*. *mykiss* was significantly longer than other species, suggesting that over centuries of domestication, this species underwent strong selection, thus its mutation rate was higher than that of other species. Genetic relationships among individuals could also be visualized in the phylogenetic tree, which could help identify subgroups within the investigated populations, as well as select proper propagative parents with enough genetic distances.

In most hatcheries in China, salmonid fishes are cultured in nearby areas because these have similar environmental conditions. As a universal genetic tool, the 57K SNP array may facilitate genetic analysis these poly-cultured salmonid species. For different purposes and species, suitable sets of SNP markers from the 57,501 candidate SNPs could be selected for genotyping. In this work, we presented the genotyping results of 96 individuals from seven populations, which belonged to four genera and seven species. Fig 1 shows that individuals from four genera shared 28,882 informative SNPs and 525 polymorphic SNPs, which in turn may be utilized in the development of a medium-density SNP array for common aquaculture salmonid fishes. The observed sharp decrease in the number of polymorphic SNPs may be due to poor genetic diversity in the tested OK populations. The full list of the 28,882 shared SNPs is shown in S1 Table, which could be used for various population genetic analyses such as PCA. The shared polymorphic SNPs could also be used as markers for pedigree assignment and propagative parent evaluation.

## Conclusions

In this study, a 57K rainbow trout SNP array was utilized to explore the genetic diversity and population relationships in a variety of salmonid populations in China. High genotyping call rates (>90%) were obtained, whereas the polymorphic rates within populations ranged from 7.8% to 96%. A number of shared SNPs across species was developed and used for population genetic analyses, including PCA and phylogenetic reconstruction. One of the study populations, *O*. *kisutch*, showed low genetic diversity. Genetic distances among individuals and subgroups within the populations were discovered. The results of the present study may be potentially used in the genetic evaluation of germplasm resources, as well as genomic selection in salmonid breeding.

## Supporting information

S1 Table Information on the 28,882 shared SNPs among salmonid species. (CSV)

S1 Fig Call rate distribution for 96 individuals from seven salmonid populations. (DOCX)
