# Supplementary material for: Population genetic analysis of aquaculture salmonid populations in China using a 57K rainbow trout SNP array
Source: PLoS One. 2018 Aug 17;13(8):e0202582. doi: 10.1371/journal.pone.0202582 (PMC6097679; doi:10.1371/journal.pone.0202582)
Supplement: S1 Fig — (DOCX) [file pone.0202582.s002.docx]

|  |  |  |  |  |  |  |  |  |  |  |  |  |  |  |
| --- | --- | --- | --- | --- | --- | --- | --- | --- | --- | --- | --- | --- | --- | --- |
|  |  | 1 | 2 | 3 | 4 | 5 | 6 | 7 | 8 | 9 | 10 | 11 | 12 |  |
|  | A | 91.55 | 91.049 | 91.751 | 91.477 | 91.404 | 92.304 | 98.183 | 98.689 | 94.953 | 98.367 | 98.783 | 98.624 |  |
|  | B | 91.471 | 92.691 | 91.819 | 90.621 | 91.583 | 91.976 | 98.741 | 95.077 | 92.224 | 98.621 | 98.758 | 98.407 |  |
|  | C | 90.991 | 92.487 | 91.811 | 91.369 | 91.273 | 93.27 | 98.908 | 98.875 | 95.452 | 99.05 | 98.478 | 99.078 |  |
|  | D | 91.724 | 92.371 | 91.082 | 91.452 | 92.195 | 93.218 | 98.864 | 94.077 | 95.991 | 98.842 | 98.122 | 98.644 |  |
|  | E | 90.823 | 92.704 | 91.713 | 91.285 | 91.381 | 93.216 | 98.903 | 98.896 | 98.798 | 98.261 | 98.706 | 98.897 |  |
|  | F | 91.468 | 92.673 | 91.858 | 91.106 | 91.708 | 93.339 | 95.104 | 90.258 | 98.859 | 98.623 | 98.635 | 99.056 |  |
|  | G | 91.781 | 91.145 | 91.701 | 91.251 | 92.287 | 91.99 | 98.645 | 95.365 | 98.65 | 99.038 | 98.478 | 98.697 |  |
|  | H | 91.631 | 92.174 | 90.739 | 91.911 | 91.525 | 92.781 | 98.122 | 98.626 | 97.943 | 98.955 | 98.99 | 98.776 |  |
|  |  |  |  |  |  |  |  |  |  |  |  |  |  |  |
|  |  | Call rate (%) |  |  |  |  |  |  |  |  |  |  |  |  |
|  |  |  | 91 | 92 | 93 | 94 | 95 | 96 | 97 | 98 | 99 | 100 |  |  |
|  |  |  |  |  |  |  |  |  |  |  |  |  |  |  |

**S1 Fig. Call rate distribution for 96 individuals from seven salmonid populations**

1: *Hucho taimen*; 2: *Oncorhynchus masou*; 3: *Salvelinus fontinalis*;

4: *Brachymystax lenok*; 5: *Salvelinus leucomaenis*; 6: *Oncorhynchus kisutch*;

7-12: *Oncorhynchus mykiss*
